# Cellular photo(geno)toxicity of gefitinib after biotransformation

**DOI:** 10.3389/fphar.2023.1208075

**Published:** 2023-06-07

**Authors:** Meryem El Ouardi, Lorena Tamarit, Ignacio Vayá, Miguel A. Miranda, Inmaculada Andreu

**Affiliations:** ^1^ Departamento de Química-Instituto de Tecnología Química UPV-CSIC, Universitat Politècnica de València, Valencia, Spain; ^2^ Unidad Mixta de Investigación UPV- IIS La Fe, Hospital Universitari i Politècnic La Fe, Valencia, Spain

**Keywords:** anticancer drug, metabolism, photodamage to biomolecules, photosensitized reaction, tyrosine kinase inhibitor

## Abstract

Gefitinib (GFT) is a selective epidermal growth factor receptor (EGFR) inhibitor clinically used for the treatment of patients with non-small cell lung cancer. Bioactivation by mainly Phase I hepatic metabolism leads to chemically reactive metabolites such as O-Demethyl gefitinib (DMT-GFT), 4-Defluoro-4-hydroxy gefitinib (DF-GFT), and O-Demorpholinopropyl gefitinib (DMOR-GFT), which display an enhanced UV-light absorption. In this context, the aim of the present study is to investigate the capability of gefitinib metabolites to induce photosensitivity disorders and to elucidate the involved mechanisms. According to the neutral red uptake (NRU) phototoxicity test, only DF-GFT metabolite can be considered non-phototoxic to cells with a photoirritation factor (PIF) close to 1. Moreover, DMOR-GFT is markedly more phototoxic than the parent drug (PIF = 48), whereas DMT-GFT is much less phototoxic (PIF = 7). Using the thiobarbituric acid reactive substances (TBARS) method as an indicator of lipid photoperoxidation, only DMOR-GFT has demonstrated the ability to photosensitize this process, resulting in a significant amount of TBARS (similar to ketoprofen, which was used as the positive control). Protein photooxidation monitored by 2,4-dinitrophenylhydrazine (DNPH) derivatization method is mainly mediated by GFT and, to a lesser extent, by DMOR-GFT; in contrast, protein oxidation associated with DMT-GFT is nearly negligible. Interestingly, the damage to cellular DNA as revealed by the comet assay, indicates that DMT-GFT has the highest photogenotoxic potential; moreover, the DNA damage induced by this metabolite is hardly repaired by the cells after a time recovery of 18 h. This could ultimately result in mutagenic and carcinogenic effects. These results could aid oncologists when prescribing TKIs to cancer patients and, thus, establish the conditions of use and recommend photoprotection guidelines.

## 1 Introduction

Significant medical advances in cancer treatment have been made during the past few decades. However, the drugs used for this cure have a limited therapeutic index, and often the responses are only just palliative and unpredictable (Chai et al., 2021; Sapio and Naviglio, 2022). In contrast, targeted therapy introduced more recently has less non-specific toxicities since it interferes with a specific molecular target, generally a protein with a crucial role in tumor growth or progression (Baudino, 2015; Zhong et al., 2021).

The epidermal growth factor receptors (EGFRs) are transmembrane glycoproteins consisting of an extracellular ligand-binding domain, a transmembrane domain, and an intracellular domain with tyrosine kinase activity. They regulate the cell signaling pathways, including cell growth, survival, migration, and differentiation (Normanno et al., 2006). Pathological alterations of EGFRs, including kinase-activating mutation or overexpression, may result in the appearance of different types of cancers and may promote solid tumor growth. Therefore, they are major targets for the design of anticancer agents (Normanno et al., 2006; da Cunha Santos et al., 2011; Teng et al., 2011). In this regard, tyrosine kinase inhibitors (TKIs) are of high interest due to their ability to block the kinase activity of these receptors (Hartmann et al., 2009; Yamaoka et al., 2018). They are orally active small molecules that have a favorable safety profile and can be easily combined with other forms of chemotherapy or radiation therapy (Hartmann et al., 2009; Yamaoka et al., 2018; Zhong et al., 2021).

**FIGURE 1:**
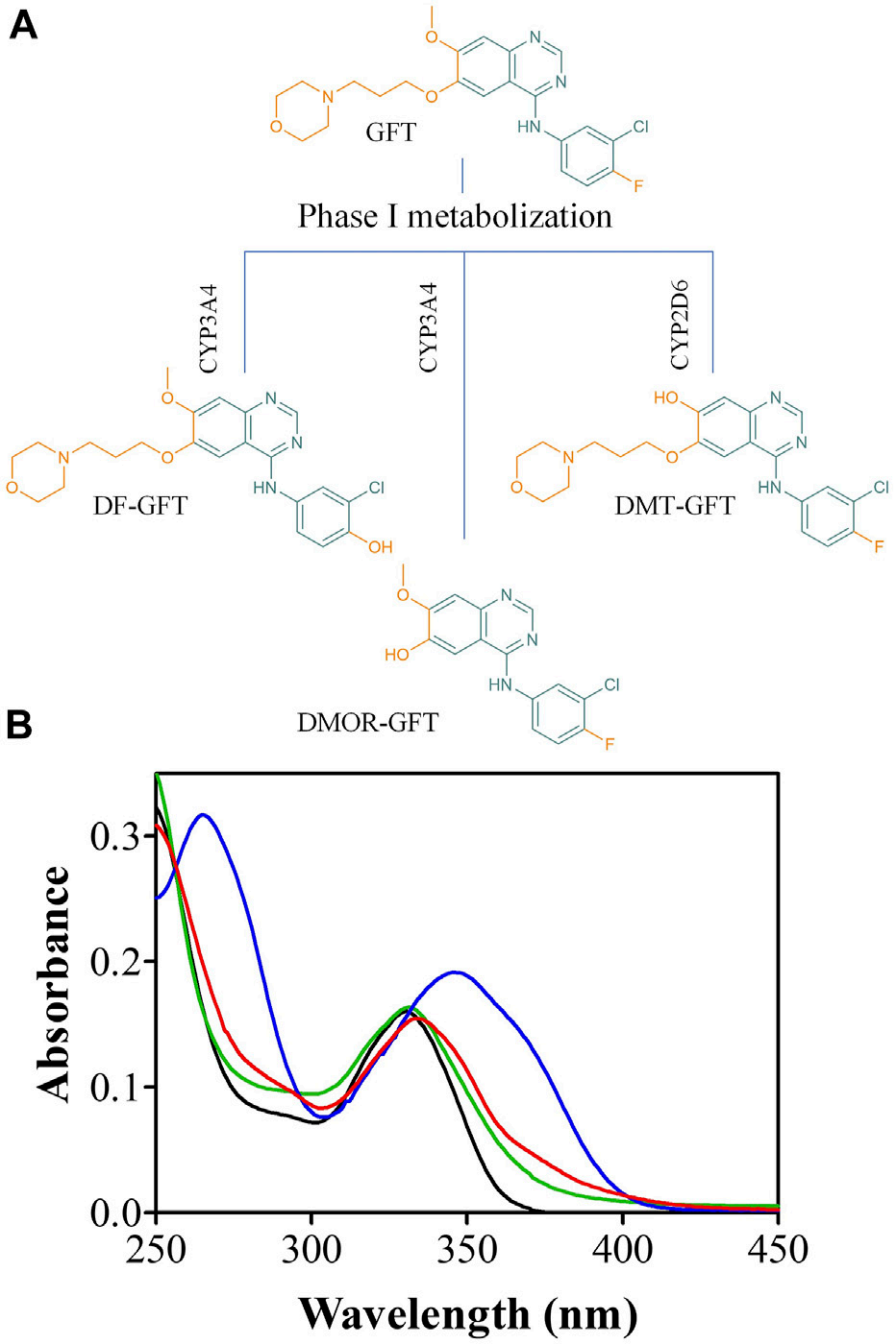
**(A)** Chemical structure of gefitinib (GFT), 4-Defluoro-4-hydroxy gefitinib (DF-GFT), O-Demethyl gefitinib (DMT-GFT) and O-Demorpholinopropyl gefitinib (DMOR-GFT); **(B)** Absorption spectra of GFT (black), DF-GFT (green), DMT-GFT (blue) and DMOR-GFT (red) in PBS at 10 µM.

In this context, it is interesting to note that TKIs are an important new class of drugs in cancer remedies (considered smart drugs) that interfere with specific cell signaling pathways and thus allow target-specific treatment for selected malignancies (Pottier et al., 2020). These anticancer drugs have significantly improved the quality of life and the survival rate of oncologic patients. Generally, they are well tolerated by patients; however, cutaneous reactions are very common, and, in most cases, enhanced by sunlight exposure (Lembo et al., 2020). Hence, although TKIs have revolutionized oncology practice over the past 20 years, very little is known about their photosensitizing potential. These side effects can be associated with damage to biomolecules mediated by radicals or reactive oxygen species arising from excited states (Salvador et al., 2014). In this context, we have previously established a good correlation between the photophysical behavior of the TKIs lapatinib and gefitinib and their photobiological properties (Vayá et al., 2020; García-Lainez et al., 2021; Tamarit et al., 2021). Gefitinib (GFT) is a selective EGFR inhibitor clinically used for the treatment of patients with non-small cell lung cancer (NSCLC) (Rawluk and WallerGefitinib, 2018). In humans, GFT undergoes metabolic bioactivation by mainly Phase I hepatic metabolism (cytochromes CYP3A4 and CYP2D6) resulting in chemically reactive metabolites such as O-Demethyl gefitinib (DMT-GFT), 4-Defluoro-4-hydroxy gefitinib (DF-GFT), and O-Demorpholinopropyl gefitinib (DMOR-GFT) (Figure 1A) (McKillop et al., 2004; Li et al., 2007; Tan et al., 2020). This chemical change generates non-negligible modifications in the quinazoline chromophore, leading to a more efficient UV-light absorption (Figure 1B). Thus, although drug biotransformation is usually regarded to a decreased toxicity, in some cases metabolites may display more phototoxicity and photoreactivity than the parent drug (Garcia-Lainez et al., 2018; Agúndez et al., 2020). Accordingly, investigating the photo(geno)toxicity of gefitinib and its metabolites is important for assessing drug safety, evaluating the risks associated with sunlight exposure during treatment. In connection with this, we have previously reported the photo(geno)toxicity induced by one of the photoactive metabolites of lapatinib, another tyrosine kinase inhibitor (Vayá et al., 2020; García-Lainez et al., 2021).

Therefore, it appears very interesting to explore the photosensitizing potential of GFT metabolites by evaluating their photo(geno)toxicity through *in vitro* studies in human skin cells to recommend preventive health measures and thus, minimize the photosensitizing risk from TKIs. Moreover, this investigation will aid the oncologists in having a better knowledge of the photoinduced adverse effects of these drugs before prescribing TKIs to cancer patients and, thus, give them photoprotection guidelines.

## 2 Materials and methods

### 2.1 Chemicals

All solvents and chemicals were commercially available (HPLC grade) and used without additional purification. Chlorpromazine hydrochloride (CPZ; CAS 69-09-0), sodium dodecyl sulfate (SDS; CAS 151-21-3), anthracene (ANT; CAS 120-112-7) and (S)-(+)-Ketoprofen (KP; CAS 22161-81-5) were purchased from Sigma-Aldrich (Madrid, Spain). Gefitinib hydrochloride (GFT; CAS 184475-55-6) was provided by MedChemExpress (New Jersey, United States). 4-Defluoro-4-hydroxy gefitinib (DF-GFT; CAS 847949-50-2) and O-Demorpholinopropyl gefitinib (DMOR-GFT; CAS 184475-71-6) were acquired from Santa Cruz Biotechnology (Dallas, United States) and O-Demethyl gefitinib (DMT-GFT; CAS 847949-49-9) was purchased from Toronto Research Chemicals (North York, Canada). Stock solutions were prepared in DMSO as vehicle, whereas GFT, CPZ and SDS were dissolved in ultrapure water (Milli-Q^®^). 1,4-dihydro-1,2-dimethylbenzoic acid (DMBA) was obtained from a Birch reduction synthesis. Dulbecco’s Modified Eagle Medium (DMEM, low glucose with pyruvate and glutamine), Dulbecco’s Modified Eagle Medium (DMEM, low glucose with pyruvate and without glutamine and phenol red), fetal bovine serum (FBS), penicillin–streptomycin (1.0 U/mL × 10^5^ U/mL, 1.0 μg/mL × 10^5^ μg/mL) and ethylenediaminetetraacetic acid (EDTA) were supplied by Honeywell Fluka (North Carolina, United States). Trypsin–EDTA (0.25%–0.02%) was provided by Cultek (Madrid, Spain). Phosphate buffered saline solution (PBS, pH 7.4), neutral red dye, human serum albumin fatty acid free (HSA), polyoxyethylenesorbitan monolaurate (TWEEN 20) and sodium hydroxide (NaOH) were purchased from Sigma-Aldrich (Madrid, Spain). Low melting point agarose was provided by Pronadisa (Madrid, Spain). Tris(hydroxymethyl)aminomethane, Linoleic acid (LA), Methyl Linoleate (ML), 1,1,3,3-Tetraethoxypropane (TEP), 2-Thiobarbituric acid (TBA) and 2,6-Di-tert-butyl-4-methylphenol (BHT) were supplied by Sigma-Aldrich (Madrid, Spain). CometAssay^®^ Lysis Solution was purchased from R&D systems (Minneapolis, United States). 2,4-Dinitrophenylhydrazine Hydrochloride (DNPH) was acquired from Santa Cruz Biotechnology (Dallas, United States). Trichloroacetic acid (TCA) was purchased from Labbox (Barcelona, Spain). SYBR™ Gold DNA, and CellMask™ Orange Plasma membrane stains were acquired from Invitrogen (Madrid, Spain). RedDot™ Far-Red Nuclear was supplied by Biotium (California, United States). Apo-ONE^®^ Homogeneous Caspase-3/7 assay and CytoTox-ONE™ Homogeneous Membrane Integrity Assay were received from Promega (Madison, United States). Deoxyribonucleic acid sodium salt from calf thymus was purchased from Sigma-Aldrich (Madrid, Spain).

### 2.2 Spectroscopic measurements

#### 2.2.1 Absorption and emission spectra measurements

Absorption spectra were recorded in a JASCO V-760 spectrophotometer using 10 × 10 mm^2^ quartz cuvettes at room temperature. For fluorescence emission experiments, solutions of GFT and metabolites in PBS were incubated in the presence of human serum albumin (HSA), methyl linoleate (ML), calf thymus DNA (ctDNA) in a ratio of 1:1 or HaCaT cells (2 × 10^4^ cells/well) in black 96-well plates for 1 h. Fluorescence spectra (*λ*_exc_ = 320 nm) were recorded using a Synergy H1 multi-mode microplate reader.

#### 2.2.2 Quenching experiments by laser flash photolysis

A pulsed Nd:YAG L52137 V LOTIS TII laser (Sp Lotis Tii, Minsk, Belarus) was used for the excitation at 355 nm. The single pulses were ∼10 ns of duration, and the energy was ∼12 mJ/pulse. Laser flash photolysis (LFP) equipment consisted of a pulsed laser, a 77250 Oriel monochromator, and an oscilloscope DP04054 Tektronix. The output signal from the oscilloscope was transferred to a personal computer for processing. The DMOR-GFT metabolite was dissolved in acetonitrile up to an absorbance of 0.3 at 355 nm. Solutions were deaerated by bubbling nitrogen through the solution during 15 min. The rate constant of triplet excited-state quenching by 1,4-dihydro-1,2-dimethylbenzoic acid (DMBA) was determined using the Stern–Volmer Equation:

$$1/\tau =1/\tau_{q}+k_{q}\;\left[DMBA\right]$$

Where *τ* and *τ*_q_ are the lifetime of transient species in the presence and absence of DMBA, respectively. Concentrations between 0.1 and 10 mM were used for DMBA. This compound was prepared through Birch reduction following standard procedures (Andreu et al., 2011).

### 2.3 Cell culture conditions

Human keratinocytes cells (HaCaT) were grown in 75 cm^2^ plastic flasks in DMEM supplemented with 10% FBS and penicillin/streptomycin (100 U/mL, and 100 μg/mL) in a humidified incubator (100% relative humidity) at 37°C with 5% CO_2_ atmosphere. Cells were routinely passed twice a week for maintenance (1:5 splitting ratio) and a trypan blue exclusion test was used to ensure that the cultures were viable before each experiment.

### 2.4 Cellular localization by confocal microscopy

HaCaT cells were seeded on sterile round glass coverslips and incubated in 24-well plates (2.5 × 10^4^ cells/well). After 24 h of incubation, DMEM medium was replaced with 1 mL of fresh medium containing drugs (GFT, DMT-GFT or DMOR-GFT) at 15 μM followed by staining with CellMask™ Orange Plasma membrane and RedDot™ Far-Red nuclear stains (1:10,000 and 1:200 dilutions, respectively). Cells treated with the compounds were incubated for 1 h whereas CellMask™ Orange Plasma membrane and RedDot™ Far-Red nuclear stains were incubated for 30 and 10 min respectively at 37°C. Then, coverslips were washed three times for 5 min with PBS and finally mounted in glass slides using a solution of mowiol. Through sequential mode, a Leica SP5 confocal microscope was used for cell imaging. The excitation wavelengths were 405 nm for GFT, DMT-GFT and DMOR-GFT. For CellMask™ Orange Plasma membrane and RedDot™ Far-Red nuclear stains, the excitation wavelengths were 543 nm and 662 nm, respectively. The maxima emission wavelengths were 450, 567, and 694 nm for the drug and its metabolites, plasma membrane and nuclear stains, respectively.

### 2.5 Irradiation equipment

Irradiations under UVA light conditions were performed with an LCZ-4 photoreactor equipped with six top and eight sides Hitachi lamps (*λ*_max_ = 350 nm, Gaussian distribution; Luzchem, Canada), which emit 94% UVA and 2% UVB radiation, respectively. The samples for *in vitro* HaCaT NRU phototoxicity assay were irradiated in 96-well transparent plates, for comet assay 24-well transparent plates were used while in both protein and lipid photooxidation assay irradiations were performed in 6-well transparent wells. All irradiations were carried out through the lid of the plates which does not absorb beyond 310 nm for the purpose of reducing the direct effect of UVB radiation over the cell cultures. Since cell viability after irradiation was higher than 90%, the UV dose selected was suitable for the photogenotoxicity experiments, therefore, false-positive results triggered by DNA fragmentation as a result of cell death were avoided. Moreover, in order to prevent overheating, plates were kept on ice during the irradiation process and the temperature was controlled by ventilation.

### 2.6 *In Vitro* neutral red uptake (NRU) phototoxicity test

Neutral red uptake phototoxicity test (NRU) was assessed according to the OECD Guideline 432 (OECD, 2019) in keratinocyte cells (HaCaT) with additional minor modifications (Garcia-Lainez et al., 2018). Chlorpromazine (CPZ) and sodium dodecyl sulfate (SDS) served as the positive phototoxic and negative non-phototoxic controls, respectively. CPZ is a widely used typical antipsychotic drug with well-known phototoxic properties as already reported (Palumbo et al., 2016). In our recent study, GFT has shown a very significant phototoxic potential in HaCaT cells (Tamarit et al., 2021). Therefore, the phototoxicity behavior of the metabolites was studied in parallel with the parent drug.

Concisely, for each compound, two 96-well plates were seeded at a density of 2.0 × 10^4^ cells/well. Next day, HaCaT cells in a fresh DMEM medium without phenol red were treated with the compounds (GFT, DF-GFT, DMT-GFT and DMOR-GFT) at eight concentrations ranging from 2.5 to 500 μM except for DMOR-GFT which was added in a concentration range from 125 to 0.625 μM. Additional plates were processed with CPZ (from 1.57 to 500 µM) and SDS (from 3.13 to 500 µM). All plates were incubated for 1 h in dark conditions. Then, for each sample, in the presence of ice, one plate was irradiated with a non-cytotoxic dose of 5 J/cm^2^ UVA (UVA Light), whereas the other was kept in a dark box (Dark). Next, drug solutions were replaced with fresh DMEM medium, and plates were further incubated overnight. Next day, neutral red solution (50 μg/mL) was added into the wells and incubated for 2 h at 37°C. Later, cells were washed once with PBS and neutral red was recovered from lysosomes in 100 μL of the extraction buffer [distilled water 50% (v/v), ethanol 49.5% (v/v) and acetic acid 0.5% (v/v)]. Lastly, the absorbance of the plates was read at 550 nm on a Synergy H1 microplate reader. For each compound, dose–response curves were performed to establish the concentration causing a reduction of 50% of neutral red uptake (IC_50_) in dark and UVA light conditions. Afterwards, photoirritation factor (PIF) values were calculated using the following equation:

$$PIF={{IC}_{50}\;\left(Dark\right)\;/\;IC}_{50}\;\left(UVA\;Light\right)$$

Conforming to OECD Guideline 432 (OECD, 2019), a substance is labelled as “non-phototoxic” when PIF is <2, “probably phototoxic” if PIF is between 2 and 5 and “phototoxic” if PIF is >5.

### 2.7 Photosensitized lipid peroxidation

Linoleic acid photosensitized oxidation assay was performed as described previously (Seto et al., 2013) with minor adjustments exposed in prior studies (Zeb and Ullah, 2016). For this, a solution of linoleic acid (1 mM) in 20 mM PBS (pH 7.4) containing 0.05% Tween 20 was prepared and irradiated in the presence of GFT or its metabolites (DMT-GFT or DMOR-GFT) in a concentration of 100 µM. KP 200 µM was taken as a positive control (Seto et al., 2013). Lipid peroxidation was monitored with TBA-reactive substances assay (TBARS) (Zeb and Ullah, 2016) adding 4 mM TBA and 10 µL BHT solution in glacial acetic acid to the irradiated samples (500 µL). Then, samples were heated at 95 °C for 60 min. After 10 min of cooling, the absorbance of the samples was measured at 532 nm for the determination of TBARS. A standard curve of TEP was used to determine the total of malondialdehyde (MDA) produced. The data was analyzed statistically using a Two-way ANOVA (Analysis of Variance) technique.

### 2.8 Photoinduced protein oxidation assay

Protein photooxidation assay was assessed according to Colombo et al. (2016) with minor modifications as explained below. The photooxidative activity of GFT has already been evaluated in our previous study (Tamarit et al., 2021), which has proved its protein photooxidation capability. Briefly, a solution of HSA (5 mg/mL, 1 mg protein/sample) was prepared in PBS and irradiated alone or in the presence of 100 μM of GFT, DMT-GFT or DMOR-GFT with a UVA dose of 15 J/cm^2^. The amount of HSA oxidation in each sample was determined immediately after irradiation by incubating the samples for 60 min at room temperature with 200 µL of 2,4-dinitrophenylhydrazine (DNPH) 10 mM to create stable protein-DNP hydrazone adducts. After incubation, proteins were precipitated with 20% TCA solution and incubated on ice for 15 min. Next, samples were washed twice with ethanol/ethyl acetate 1:1 (v/v) containing 20% TCA, in order to reduce the protein loss, followed by its resolubilization in 100 μL guanidine buffer (6 M). Finally, absorbance at 375 nm was registered using the Synergy H1 microplate reader and the HSA oxidation degree was expressed as nmol of carbonyl per mg protein as displayed in the next equation:

$$Carbonyl\;content\;\left(nmol/mg\;protein\right)=\frac{\left(A_{375}/\varepsilon^{mM}\right)}{P}\times 100$$

Where A_375_ is the absorbance of the sample at 375 nm, ε^mM^ is the corrected millimolar extinction coefficient (6.364) and P the amount of protein from standard well.

The statistical analysis of the data was conducted using a Two-way ANOVA (Analysis of Variance) method.

### 2.9 Nuclear DNA damage by single cell gel electrophoresis (comet) assay

The comet assay (single-cell gel electrophoresis) was performed as already detailed in Garcia-Lainez et al. (2018) in order to detect both single and double strand breaks and alkaline labile sites on nuclear DNA. Briefly, HaCaT cells in exponential growth were harvested by trypsinization and kept in ice-cold PBS for 2 h to neutralize any DNA damage produced during trypsinization step, as has been previously demonstrated in FSK cells (Agúndez et al., 2020). Then, 24-wells plates (1.0 × 10^4^ cells/well) were seeded and treated with 100 μM of GFT or its metabolites (DMT-GFT or DMOR-GFT) for 30 min at 4°C in darkness to minimize cell aggregation and inhibit DNA repair. The reference photogenotoxic control of this assay was CPZ (10 μM). Afterwards, one plate was irradiated under 2 J/cm^2^ UVA light dose (4 J/cm^2^ for DMT-GFT and DMOR-GFT) while the other one was kept in darkness as the negative control. Later, both irradiated and non-irradiated cells were detached from the plates and 100 µL of cell suspension was mixed with 100 µL 1% low melting point agarose solution and loaded onto FLARE^®^ slides. Then, slides were incubated on ice to allow drop jellification. Finally, slides were embedded in a box with lysis buffer to initiate the lysis of cells and incubated overnight at 4°C. Next day, slides were placed in electrophoresis tank filled with 1 L of cold alkaline electrophoresis buffer (0.2 M NaOH, 1 mM EDTA in distilled water and pH ≥ 13). The electrophoresis was run at 21 V (1 V/cm) for 30 min and kept at 4°C. Then, slides were washed twice with MilliQ water and DNA fixation accomplished by a serial dehydration with 70% ethanol and 100% ethanol solutions during 5 min and followed by drying for 2 h at 37°C. Nuclear DNA was stained with a SYBR Gold^®^ bath (1:10,000 in Tris-EDTA buffer) for 30 min at 4°C and slides were kept in darkness until its visualization. Comets (nucleoids and tails) were visualized using a fluorescence microscope (Leica DMI 4000B). DNA damage was quantified by counting and analyzing at least 100 DNA comets. Finally, total comet score (TCS) was obtained with the classification of six DNA damage types (Møller, 2006) applying the formula:

$$\left[\left(Cl.\;0\times 0\right)+\left(Cl.\;1\times 1\right)+\left(Cl.\;2\times 2\right)+\left(Cl.\;3\times 3\right)+[\left(Cl.\;4\times 4\right)+\left(Cl.\;5\times 5\right)+\left(Cl.\;6\times 6\right)]/6\right.$$

Where Cl. is the class of DNA damage according to the visual scoring.

The results are expressed in 1–100 arbitrary units, where class 0 comets are comets with no DNA damage while class 6 comets represent comets with maximum DNA damage. Two-way ANOVA (Analysis of Variance) method was used for the statistical analysis of the data.

### 2.10 Measurement of cell death

#### 2.10.1 Caspase 3/7 activity assay

HaCaT cells were seeded in 96-well plates at a concentration of 2 × 10^5^ cell/mL. The next day, cells, in fresh DMEM medium without phenol red, were treated with the compounds at two different concentrations representing 100% and 50% of cell viability according to NRU dose-response curves. After 1 h incubation at 37°C, one plate was irradiated with UVA light (5 J/cm^2^) and the other one was kept in dark conditions. Later, plates were incubated for another 24 h at 37°C with fresh DMEM medium. Then, samples were analyzed by the Apo-ONE Homogeneous Caspase-3/7 assay according to the manufacturer’s instructions. Immediately after, the fluorescence was monitored at 0 h and 4 h after the addition of the substrate using Synergy H1 multi-mode microplate reader (excitation at 499 nm and emission at 521 nm). The data was subjected to statistical analysis using the Two-way ANOVA (Analysis of Variance).

#### 2.10.2 Lactate Dehydrogenase release assay

Lactate Dehydrogenase activity assay was performed according to the manufacturer´s protocol. Briefly, HaCaT cells were seeded in 96-well plates (2 × 10^5^ cell/mL) and incubated for 24 h. Then, after replacement of the medium with fresh no-phenol red DMEM, cells were incubated for 1 h with the compounds in two different concentrations representing 100% and 50% of cell viability according to NRU dose-response curves. Next, one plate was irradiated with UVA light (5 J/cm^2^) while the other one was kept in dark conditions. Next day, samples were analyzed by CytoTox-ONE™ Homogeneous Membrane Integrity Assay and a lysis solution (9% weight/volume solution of Triton X-100 in water) was used to determine the maximum amount of LDH present. The fluorescence was measured 10 min after the addition of the substrate using Synergy H1 multi-mode microplate reader (excitation at 560 nm and emission at 590 nm). The percent of LDH release was determined following the following equation:

$$\%\;LDH\;release=\frac{Experimental\;LDH\;release}{Maximum\;LDH\;release}\;x\;100$$

The data was analyzed statistically employing the Two-way ANOVA (Analysis of Variance) method.

### 2.11 Data analysis and statistics

Results are presented as mean ± standard deviation retrieved from the results of at least three independent experiments. Data were analyzed and regression methods were developed using either GraphPad or OriginLab software. Statistical significance was obtained from the Student’s t-test and *p* values lower than 0.05 were considered significant (*p < 0.05; **p < 0.01; ***p < 0.001).

## 3 Results and discussion

### 3.1 Phototoxicity of gefitinib metabolites

*In vitro* phototoxicity testing enables the early detection and screening of substances or compounds that may exhibit phototoxic properties. Thus, in a first stage, *in vitro* neutral red uptake (NRU) phototoxicity studies were performed with HaCaT cells to determine the phototoxic potential of GFT and its metabolites upon exposure to UVA light (5 J/cm^2^).

#### 3.1.1 *In vitro* neutral red uptake phototoxicity test

Viability of HaCaT cells was measured after treatment with the compounds employing as vital dye the neutral red, both in darkness and in the presence of UVA light. Non-linear regression dose-response curves were obtained and IC_50_ values were determined (Supplementary Figure S1). Then, the photoirritation factor (PIF) value was calculated as a ratio between the IC_50_ with and without UVA irradiation for GFT and its metabolites. The obtained values are collected in Table 1. As already found in our previous study, GFT is undoubtedly phototoxic with a PIF value of 13. The demethylated metabolite (DMT-GFT) showed a decrease in the phototoxic potential; however, following the OECD 432 Guide (OECD, 2019), DMT-GFT can still be considered phototoxic. Conversely, replacement of the fluorine substituent with OH (DF-GFT) resulted in a negligible phototoxic activity. Surprisingly, dealkylation of the propoxy-morpholine side chain (DMOR-GFT) led to a notable enhancement of the phototoxic potential with a PIF value as high as 48.

**TABLE 1:** *In vitro* HaCaT NRU phototoxicity assay of GFT and its metabolites^a^.

Compound	IC_50_ dark (µM)	IC_50_ UVA light (µM)	Photoirritant factor (PIF)^b^
CPZ	80 ± 17	4 ± 0.9	20
GFT^c^	64 ± 3	5 ± 1.7	13
DF-GFT	1,015 ± 222	1,099 ± 406	1
DMT-GFT	34 ± 3	5 ± 0.1	7
DMOR-GFT	144 ± 6	3 ± 0.6	48
SDS	133 ± 31	136 ± 23	1

^a^ Data represent the mean ± SD from 4 independent dose-response curves. CPZ and SDS were selected as positive and negative controls of phototoxicity, respectively.

^b^ According to the OECD 432 Guide (OECD, 2019), PIF <2 means “no phototoxicity”, 2< PIF <5 means “probable phototoxicity” and PIF >5 means “phototoxicity.”

^c^ The PIF value of GFT was taken from the literature (Tamarit et al., 2021).

#### 3.1.2 Lipid photoperoxidation

**FIGURE 2:**
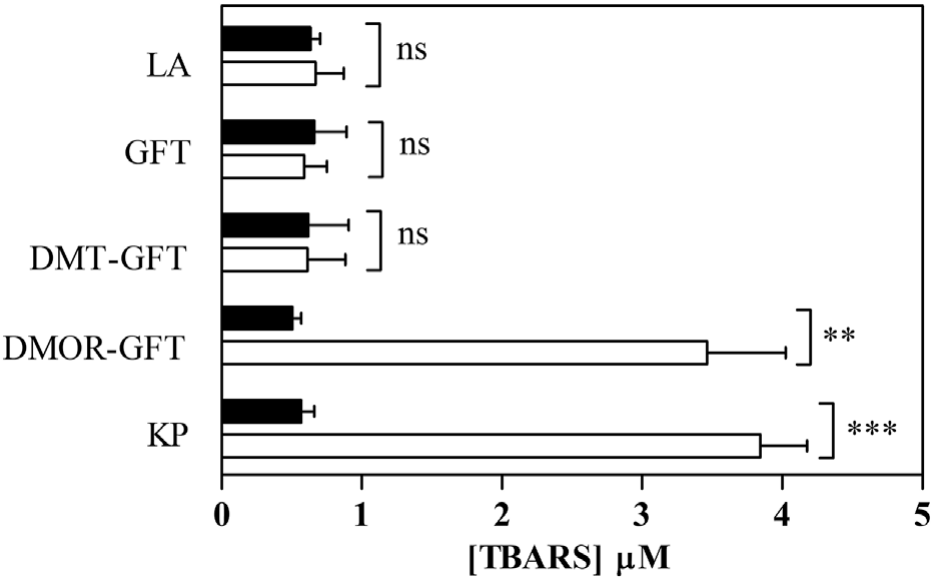
Photosensitized lipid peroxidation. Solutions of linoleic acid (LA) in PBS alone or with GFT/metabolites (100 µM) were kept in darkness (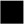 Dark) or exposed to a UVA dose of 15 J/cm^2^ (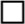 UVA Light), and TBARS content was monitored with TBA method. Ketoprofen (KP) 200 µM was selected as a reference. Data represent mean ± SD of at least 4 independent experiments. Asterisks indicate significant changes in relation to TBARS formation under darkness by the Student’s t-test (**p < 0.01, ***p < 0.001 and ns: non-significant).

Photoinduced lipid peroxidation can be one of the processes involved in cellular phototoxicity. A previous investigation has confirmed that various phototoxic drugs induce the peroxidation of linoleic acid, leading to high levels of thiobarbituric acid reactive substances (TBARS) (Onoue and Tsuda, 2006). Herein, photosensitized lipid peroxidation properties were studied for GFT and its metabolites upon UVA light irradiation in the presence of linoleic acid (1 mM). Results are given in Figure 2. According to expectations, KP (200 µM) generated a significant amount of TBARS, which confirms the suitability of this compound as a reference for photo induced peroxidation of linoleic acid. On the contrary, neither GFT nor DMT-GFT showed detectable changes in the amount of TBARS, whereas irradiation with DMOR-GFT produced a remarkable enhancement in photosensitized lipid peroxidation comparable to the reference.

**FIGURE 3:**
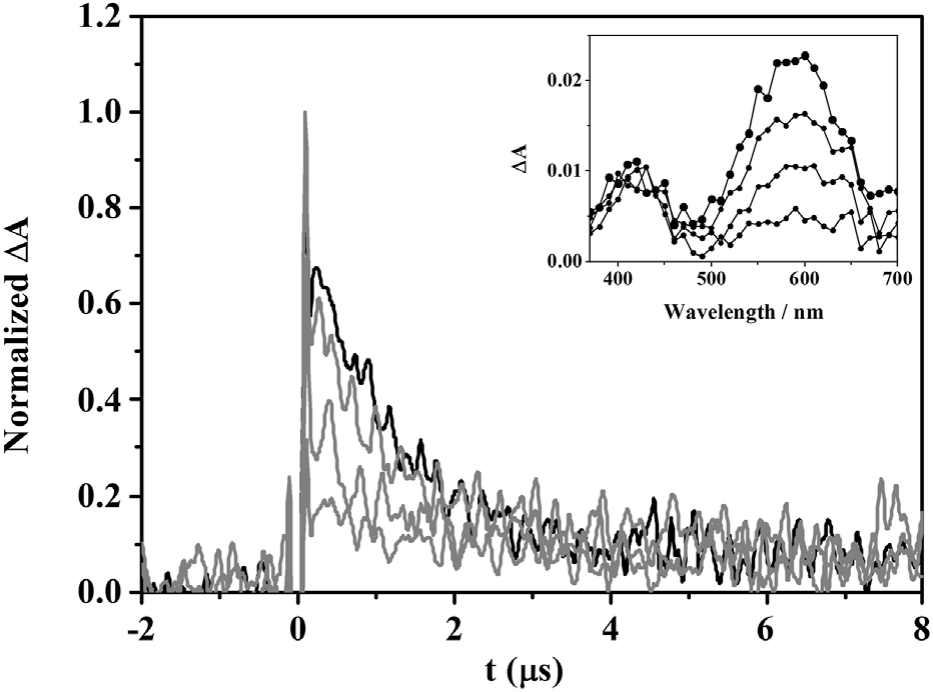
Decays at 610 nm for DMOR-GFT alone (black) or in the presence of an increasing amount of DMBA (0.1–10 mM) (gray) in deaerated acetonitrile solution after the 355 nm laser excitation. Inset: triplet-triplet absorption spectra of DMOR-GFT (from 0.2 to 3 µs).

In principle, photodynamic lipid peroxidation may occur via Type I (radical-mediated) or Type II (trough singlet oxygen, ^1^O_2_) mechanisms, where a common intermediate is the triplet excited state of the photosensitizer. In the case of GFT and DMOR-GFT, the triplet states (^3^GFT* and ^3^DMOR-GFT*) have been previously identified and characterized by means of their transient absorption at ca. 600 nm. The capability of these species to produce ^1^O_2_ was studied by time-resolved NIR emission at 1,270 nm, and the quantum yields (*Φ*_Δ_ ≤ 0.1) were found to be very low. This indicates a marginal participation of Type II process and suggests that it would be interesting to investigate the possible involvement of the Type I oxidative mechanism in the lipid peroxidation photosensitized by DMOR-GFT. For this purpose, ^3^DMOR-GFT* quenching experiments were performed using 1,4-dihydro-1,2-dimethylbenzoic (DMBA) as a lipid model, which contains double allylic hydrogens and is a suitable probe for studying the reactivity of lipids with photosensitizing drugs (Andreu et al., 2011). Thus, using the laser flash photolysis (LFP) technique, triplet decay traces were obtained in deaerated acetonitrile solutions of DMOR-GFT after the addition of increasing quencher concentration. As shown in Figure 3, the DMOR-GFT triplet species was efficiently quenched by DMBA with a rate constant (k_q_) of 1.96 M^−1^·s^−1^ × 10^9^ M^−1^·s^−1^ (Supplementary Figure S2). This is consistent with the photoreaction between DMOR-GFT metabolite and DMBA model system proceeding by a Type I mechanism.

#### 3.1.3 Protein photooxidation

**FIGURE 4:**
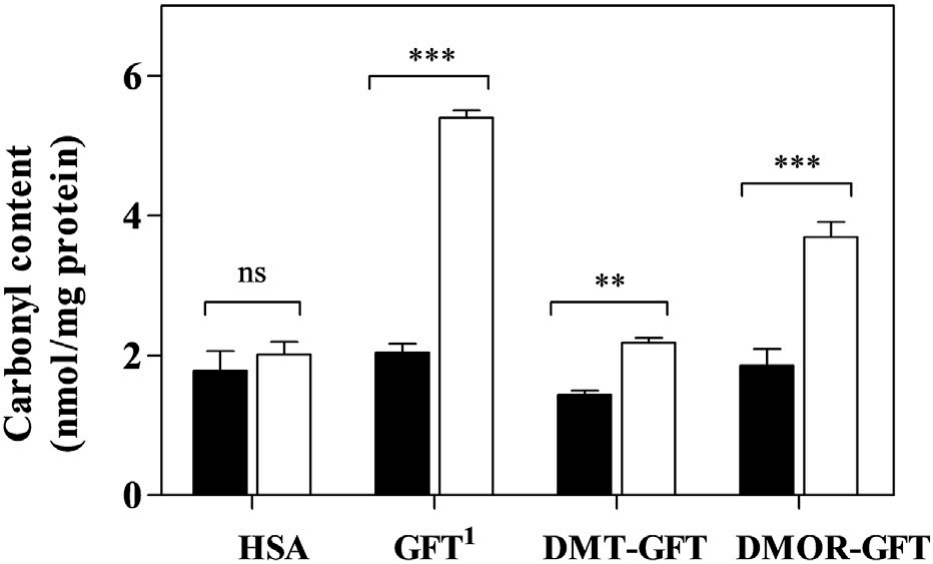
Protein carbonyl content assay. HSA solutions in PBS (5 mg/mL), alone (HSA) or in the presence of 100 μM of GFT, DMT-GFT and DMOR-GFT were irradiated with a UVA dose of 15 J/cm^2^ (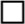 UVA Light) or kept in dark conditions (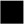 Dark). Protein oxidation was spectrophotometrically evaluated by the determination of carbonyl content with 2,4-dinitrophenylhydrazine (DNPH) derivatization assay. Data are the mean ± SD of 4 independent determinations. Asterisks indicate significant changes in relation to the carbonyl content of HSA under darkness by the Student’s t-test (**p < 0.01, ***p < 0.001 and ns: non-significant). 1, Carbonyl content value of GFT was taken from the literature (Tamarit et al., 2021).

The pharmacological target of GFT, as a tyrosine kinase inhibitor, is the ATP-rich site of plasmatic membrane receptor EGFR in cancer cells. It is known that GFT is highly protein bound in human plasma, specially to human serum albumin (HSA) (Li et al., 2006). Thus, the protein oxidation photoinduced by GFT, DMT-GFT and DMOR-GFT was studied using HSA as a model. To this end, PBS solutions of HSA and GFT, DMT-GFT or DMOR-GFT were irradiated, and then the carbonyl content, as an early biomarker of oxidative damage, was determined by the 2,4-dinitrophenylhydrazine (DNPH) derivatization method. As illustrated in Figure 4, no significant differences were found between irradiated and non-irradiated HSA, indicating the suitability of the UVA dose selected for this assay. In accordance with NRU phototoxicity results, GFT and DMOR-GFT displayed a significant oxidative effect towards HSA, although GFT showed a higher activity than the metabolite. It is interesting to recall that DMOR-GFT showed the highest PIF value among the compounds evaluated; hence, the phototoxic potential seems to be better correlated with lipid peroxidation than with protein oxidation. Lastly, DMT-GFT did not exhibit any lipid or protein oxidative damage, consistent with the NRU phototoxicity assay outcome.

### 3.2 Fluorescence properties and *in vitro* cellular uptake of GFT and its metabolites

**FIGURE 5:**
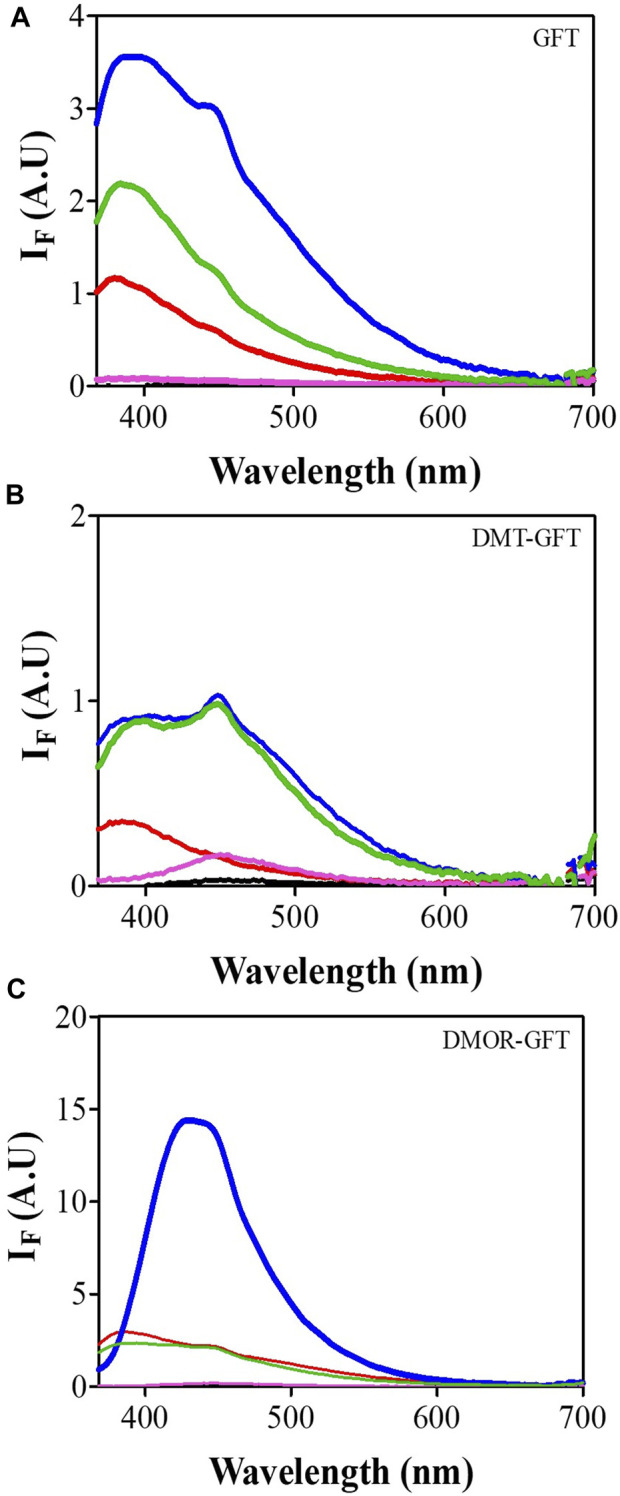
Emission spectra (*λ*_exc_ = 320 nm) of GFT **(A)**, DMT-GFT **(B)** and DMOR-GFT **(C)** alone (black) or in the presence of methyl linoleate (red), HSA (blue), ctDNA (magenta), or inside HaCaT cells (green).

Having established the phototoxic potential of GFT (Tamarit et al., 2021), DMT-GFT and DMOR-GFT (Table 1), the fluorescence spectral characteristics of these compounds were analyzed in order to investigate the photophysical differences between GFT and its metabolites in different biological media. Thus, the emission spectra (*λ*_exc_ = 320 nm) were recorded in PBS solution and in the presence of different biomolecules (lipids, protein and DNA) or inside keratinocyte cells. As displayed in Figure 5 and Table 2, an evident red shift in the emission maximum occurred for the metabolites in all models, probably due to emission from the phenolate form. Expectedly, in terms of fluorescence intensity, the protein environment increased fluorescence emission in all cases, especially for the DMOR-GFT metabolite, which was markedly enhanced. On this basis, it can be foreseen that this effect is also observed once the compounds are inside keratinocyte cells (Figure 5).

**TABLE 2:** Emission maximum wavelength of GFT and metabolites in different media^a^.

Media	Emission (λ_max_)		
	GFT	DMT-GFT	DMOR-GFT
ML^b^	380	384	384
HSA	386	394, 448	432
ctDNA^c^	390	450	448
HaCaT cells	388	392, 448	392, 448

^a^ Excitation at 320 nm.

^b^Methyl linoleate (ML).

^c^ Calf thymus DNA (ctDNA).

In parallel, fluorescence quantum yield (Φ_F_) of the internalized compounds were also determined, by comparison with anthracene as standard (*Φ*_F_ = 0.27 in ethanol) [26]. Hence, both GFT and DMT-GFT showed similar values, *Φ*_F_ = 0.07 and *Φ*_F_ = 0.04, respectively. In contrast, DMOR-GFT fluorescence inside the cells revealed a measurable enhancement of the quantum yield (*Φ*_F_ = 0.1) (Supplementary Figure S3).

**FIGURE 6:**
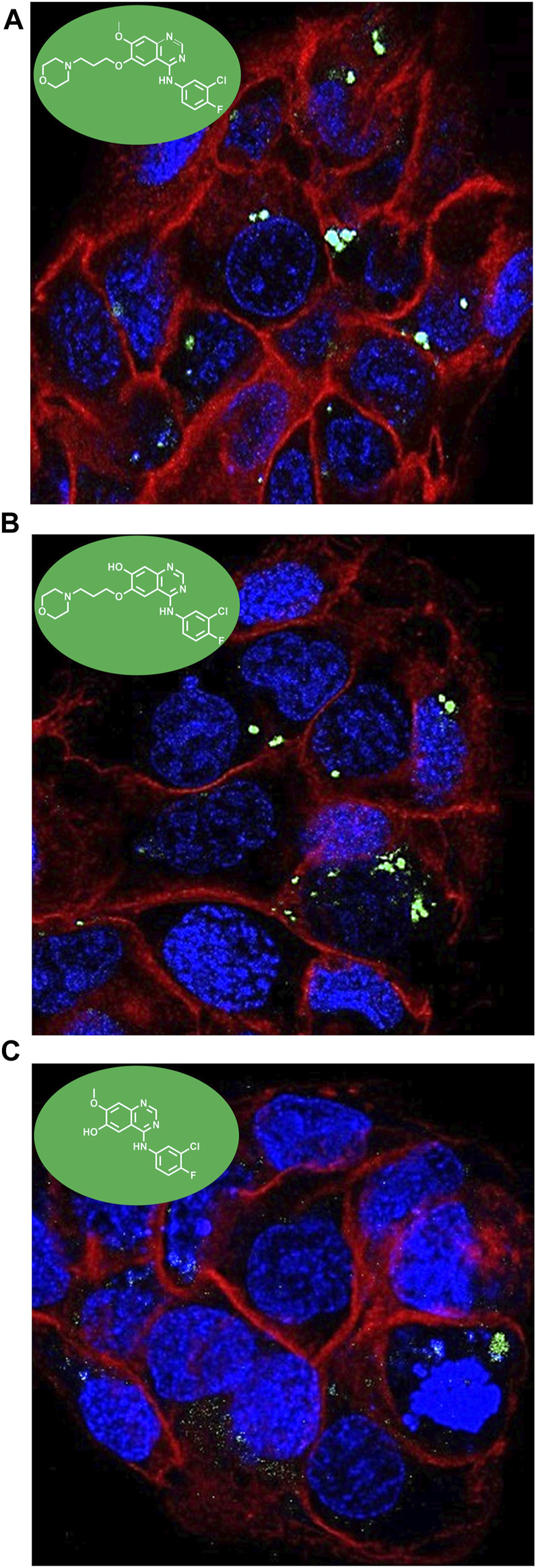
Intracellular colocalization of **(A)** GFT; **(B)** DMT-GFT and **(C)** DMOR-GFT 15 µM in HaCaT cells by confocal microscopy. A cytoplasmic distribution is observed for both drug and metabolites.

Considering the intrinsic fluorescence properties stated above, confocal microscopy was used to define the intracellular colocalization of the compounds. Keratinocytes were seeded in coverslips and treated with GFT 15 μM, DMT-GFT 25 µM and DMOR-GFT 15 µM and further labeled with both RedDot™ Far-Red Nuclear (far red fluorescence) and CellMask™ Orange Plasma membrane stains (red fluorescence). After 1 h incubation, the uptake was efficiently observed in all compounds. A cytoplasmic distribution was shown in all cases without a predominant specific localization in any organelle (Figure 6).

### 3.3 Photogenotoxicity

**FIGURE 7:**
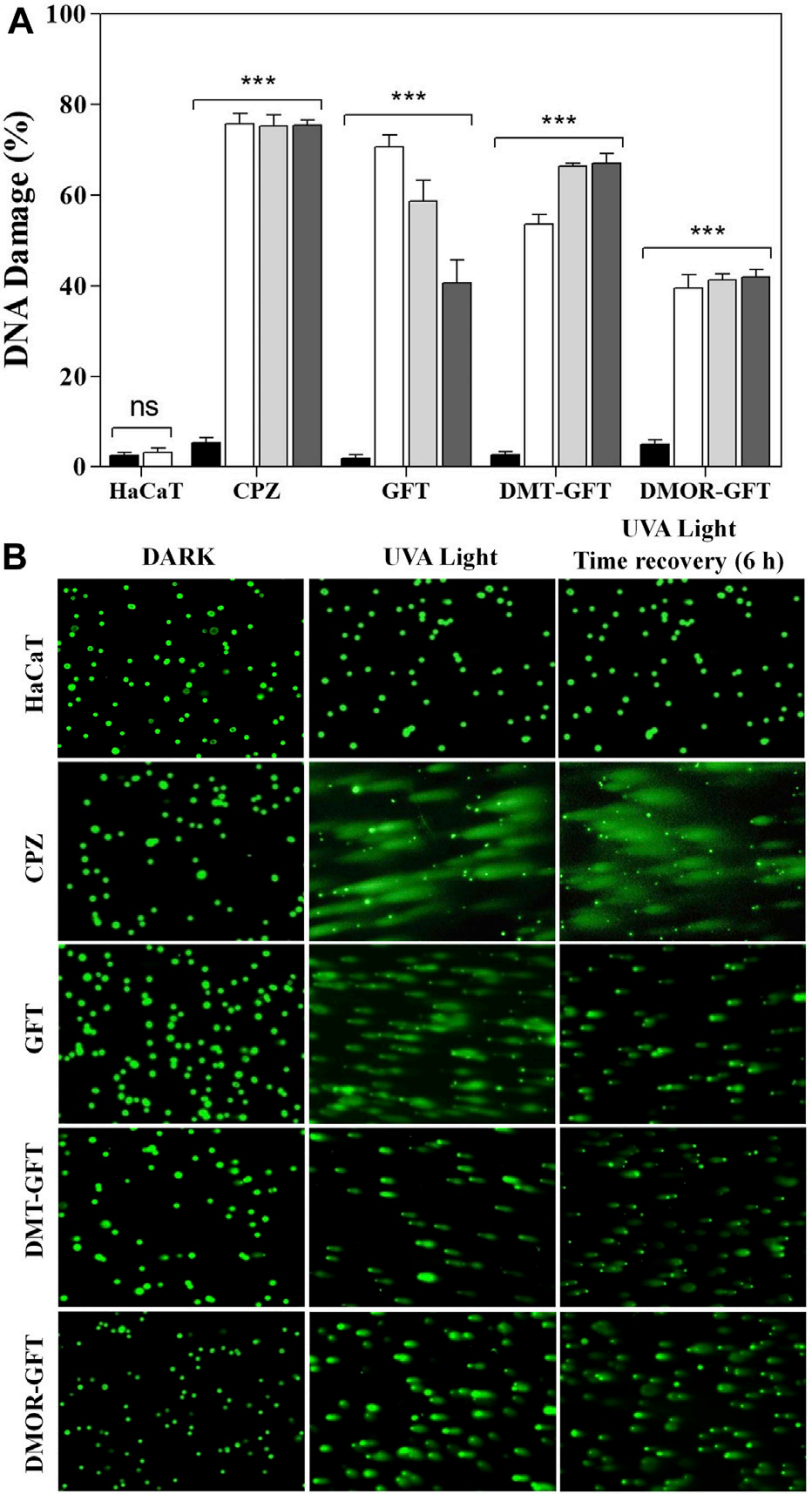
**(A)** Alkaline comet assay and DNA damage repair ability of HaCaT cells treated with 100 µM of GFT or metabolites. Cells were irradiated with UVA light at 2 J/cm^2^ dose, 4 J/cm^2^ for DMT-GFT and DMOR-GFT (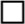), followed by 6 h (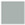) or 18 h (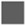) of cell recovery, or kept in the dark (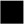). Data are displayed as the percentage of DNA damage calculated following the six visual scoring categories. Data represent the mean ± SD of 3 independent experiments. Asterisks indicate significant differences by the Student’s t-test (***p < 0.001, ns: non-significant). **(B)** Representative microscopy images of irradiated (UVA Light) or non-irradiated (Dark) cells non-treated or treated with GFT, DMT-GFT or DMOR-GFT and followed by 6 h of cell recovery (UVA Light + Time recovery 6 h).

Single-cell gel electrophoresis (comet assay) under alkaline conditions was carried out to disclose DNA damage as a result of single strand and double strand breaks as well as alkali-labile sites on chromosomic DNA of individual human keratinocytes. Hence, HaCaT cells were embedded in low melting point agarose on a slide and incubated with the compounds for 1 h. Then, samples were exposed to UVA light for 5 min (10 min for DMT-GFT and DMOR-GFT) and alkaline electrophoresis was performed after cell lysis. During electrophoresis, damaged DNA migrates from the nucleus yielding to formation of comet nucleoids and tails, which were visualized by fluorescence after SYBR Gold staining. Percentage of DNA damage was calculated following the classification of the images in six different classes. As shown in Figure 7, GFT displayed a significant damage (around 72%) after 5 min of UVA light irradiation. Likewise, DMT-GFT generated high percentage of DNA damage (around 54%). In contrast, irradiation of DMOR-GFT for 5 min did not promote DNA damage given that the nucleoids remained intact compared to control cells. Further irradiation of DMOR-GFT up to 10 min led to a significant degree of DNA damage (around 45%).

Generally, cells have evolved a number of repair mechanisms to reduce DNA damage; however, if the repair is faulty, DNA lesions can result in long-term mutations that can ultimately result in carcinogenic effects. For this reason, a complementary set of experiments were performed for the purpose of studying the capability of HaCaT cells to repair the nuclear DNA photodamage produced by GFT and its metabolites after UVA light irradiation. In brief, HaCaT cells containing GFT, DMT-GFT or DMOR-GFT were irradiated and processed for comet assay with two different incubation time (6 h and 18 h) before cell lysis was performed. Finally, DNA damage was monitored as mentioned earlier. Apparently, cells treated with GFT recovered considerably from the initial DNA damage although a residual damage was still remanent even when the recovery time reached 18 h (41%) (Figure 7). This trend was not found either with DMT-GFT or with DMOR-GFT treated cells, which maintained the initial damage intact (Figure 7). More details are provided in the supplementary information (Supplementary Figure S4).

### 3.4 Cell death mechanisms (apoptosis vs. necrosis)

**FIGURE 8:**
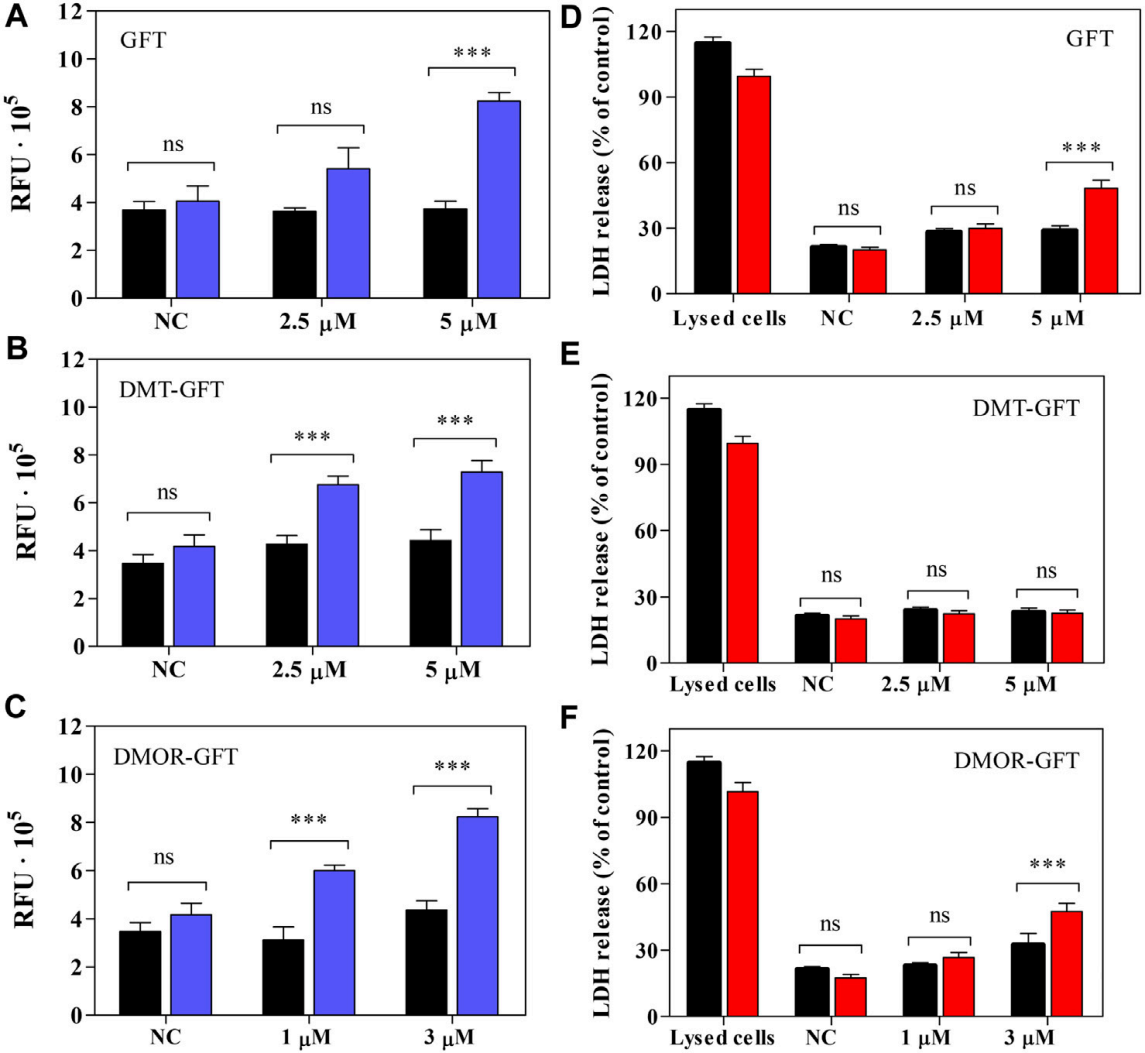
**(A–C)** The effect of GFT and its metabolites on caspase-3/7 activity in HaCaT cells before (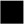) and after UVA light exposure at 5 J/cm^2^ dose (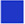). **(D–F)** Lactate dehydrogenase (LDH) release in HaCaT cells treated with GFT and metabolites before (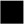) and after UVA light exposure at 5 J/cm^2^ dose (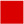). Data represent the mean ± SD of 3 independent experiments. Asterisks indicate significant differences by the Student’s t-test (***p < 0.001, ns, non-significant).

Apoptosis, frequently referred to as programmed cell death, plays a crucial role in the regulation of the cellular lifecycle (Kumar, 2007), however; excessive activation of this process can lead to critical diseases (Kam and Ferch, 2000). The apoptosis event involves, in most cases, the activation of the so-called zymogens, which are evidenced to be the precursors of the well-known caspase enzymes (Kumar, 2007). Upon apoptotic signals, caspases are activated and, through their proteolytic activity, initiate protein digest inducing cell death (Morgan and Thorburn, 2001). The key effector caspases in mammals are caspase-3, -6 and -7, being caspase-3 the most frequently involved in apoptosis (Kumar, 2007). Based on these facts, the contribution of GFT or its metabolites to apoptosis upon UVA light exposure was investigated in HaCaT cells using Apo-ONE homogeneous caspase-3/7 assay. The kit contains a profluorescent Rhodamine 110 (Z-DEVD-R110), which serves as a substrate for both caspase-3 and caspase-7. Consequent to the cleavage and removal of the DEVD peptide by caspase-3/7, Rhodamine 110 (the leaving group) becomes intensely fluorescent. Considering this, caspase-3/7 activity was monitored by fluorescence and represented as a relative change, indicating the level of apoptosis activation inside the cells. Thus, both GFT and DMOR-GFT induce a concentration dependent activation of caspase-3/7 after UVA light exposure, as shown in Figures 8A, C, specially at the concentration close to the IC_50_ value (5 µM and 3 μM, respectively). In the case of DMT-GFT (Figure 8B), caspase-3/7 activity was similar between the concentration of 2.5 µM and the concentration corresponding to IC_50_ (5 µM).

L-Lactate dehydrogenase (LDH) is a stable cytoplasmic enzyme that catalyzes the conversion of lactate to pyruvate, as it converts NAD^+^ to NADH during glycolysis (Kaja et al., 2015). LDH-release assay is used to assess the level of plasma membrane damage since the permeabilization of the membrane cause the leakage of this enzyme out of the cells (Chan et al., 2013). Bearing in mind that the key factor for necrotic cells is the permeabilization of the plasma membrane, the measurement of LDH-release can be considered an indicator for necrosis. However, it is interesting to highlight that the leakage of LDH may also be involved in apoptotic events in late stages (Parhamifar et al., 2013). As shown in Figure 8D, a small but significant percent of LDH-release was displayed for both GFT at a concentration near the IC_50_, respectively. Contrary, DMT-GFT (Figure 8E) did not show any effect on the LDH release. Additionally, in Figure 8F, a significant percentage of LDH-release is also observed.

In summary, gefitinib represents an important targeted therapy for certain types of cancer, offering personalized treatment options and potentially improved outcomes. However, it is crucial to carefully consider the photo(geno)toxic potential of both the parent drug and its metabolites.

## 4 Conclusion

Phase I biotransformation of GFT leads to reactive metabolites. This chemical event generates non-negligible modifications in the quinazoline chromophore, leading to a significant change in its light-absorbing properties. Here, it has been investigated the photobehavior of GFT and its reactive metabolites (DMT-GFT and DMOR-GFT) towards biomolecules (lipids, proteins and DNA) as well as in cellular milieu using human keratinocytes. The metabolite DMOR-GFT is markedly more phototoxic to cells than the parent drug, according to the NRU *in vitro* studies, whereas DMT-GFT is much less phototoxic. As regards the photosensitized lipid peroxidation, only DMOR-GFT is clearly effective in the TBARS assay; the weak production of singlet oxygen, combined with the efficient triplet excited state quenching by a lipid model containing double allylic hydrogens, support the involvement of a Type I mechanism. Protein photooxidation (monitored by carbonyl content measurements) is mainly mediated by GFT and, to a lesser extent, by DMOR-GFT; in contrast, protein oxidation associated with DMT-GFT is nearly detectable. This reaction is explained by initial electron transfer from the oxidizable amino acid residues to the quinazoline moiety. Damage to cellular DNA, as revealed by the comet assay, occurs upon irradiation in the presence of the parent GFT and its two metabolites. Interestingly, the most efficient photosensitizer in this case is DMT-GFT; moreover, the DNA damage induced by this metabolite is hardly repaired by the cells after several hours.

Overall, the observed cellular phototoxicity can be satisfactorily correlated with the results from the mechanistic studies. Thus, DMOR-GFT, which displays the highest phototoxicity, produces the most remarkable lipid photoperoxidation and is also significantly active in the protein oxidation and DNA damage studies. Conversely, DMT-GFT is the weakest phototoxic, but it shows the highest photogenotoxicity in the comet assay. The parent drug GFT constitutes an intermediate case. Hence, cellular phototoxicity seems to be rather related to photooxidation of membrane components through a Type I (radical-mediated) mechanism. These findings highlight that the biotransformation of the anticancer drug gefitinib leads to a double-edged sword cellular photo(geno)toxicity. This knowledge is crucial for the development of new TKIs to anticipate and mitigate potential phototoxic side effects. All these considerations have to be taken into account by oncologists when prescribing TKIs to cancer patients, in order to establish the conditions of use and to recommend photoprotection guidelines.

## Data Availability

The original contributions presented in the study are included in the article/Supplementary Material, further inquiries can be directed to the corresponding authors.
