# Supplementary material for: Cellular photo(geno)toxicity of gefitinib after biotransformation
Source: Front Pharmacol. 2023 Jun 7;14:1208075. doi: 10.3389/fphar.2023.1208075 (PMC10283009; doi:10.3389/fphar.2023.1208075)
Supplement: Supplementary file 1 [file DataSheet9.PDF]

Supplementary Material

Cellular photo(geno)toxicity of the anticancer drug gefitinib after biotransformation

Meryem El Ouardi[1,2], Lorena Tamarit[1,2], Ignacio Vayá[1,2], Miguel A. Miranda[1,2]*, Inmaculada Andreu[1,2]*

[1]Departamento de Química-Instituto de Tecnología Química UPV-CSIC. Universitat Politècnica de València, Camino de Vera s/n, 46022, Valencia, Spain

[2]Unidad Mixta de Investigación UPV- IIS La Fe, Hospital Universitari i Politècnic La Fe, Avenida de Fernando Abril Martorell 106, 46026, Valencia, Spain

*** Correspondence:**
Miguel A. Miranda: mmiranda@qim.upv.es
Inmaculada Andreu: iandreur@qim.upv.es

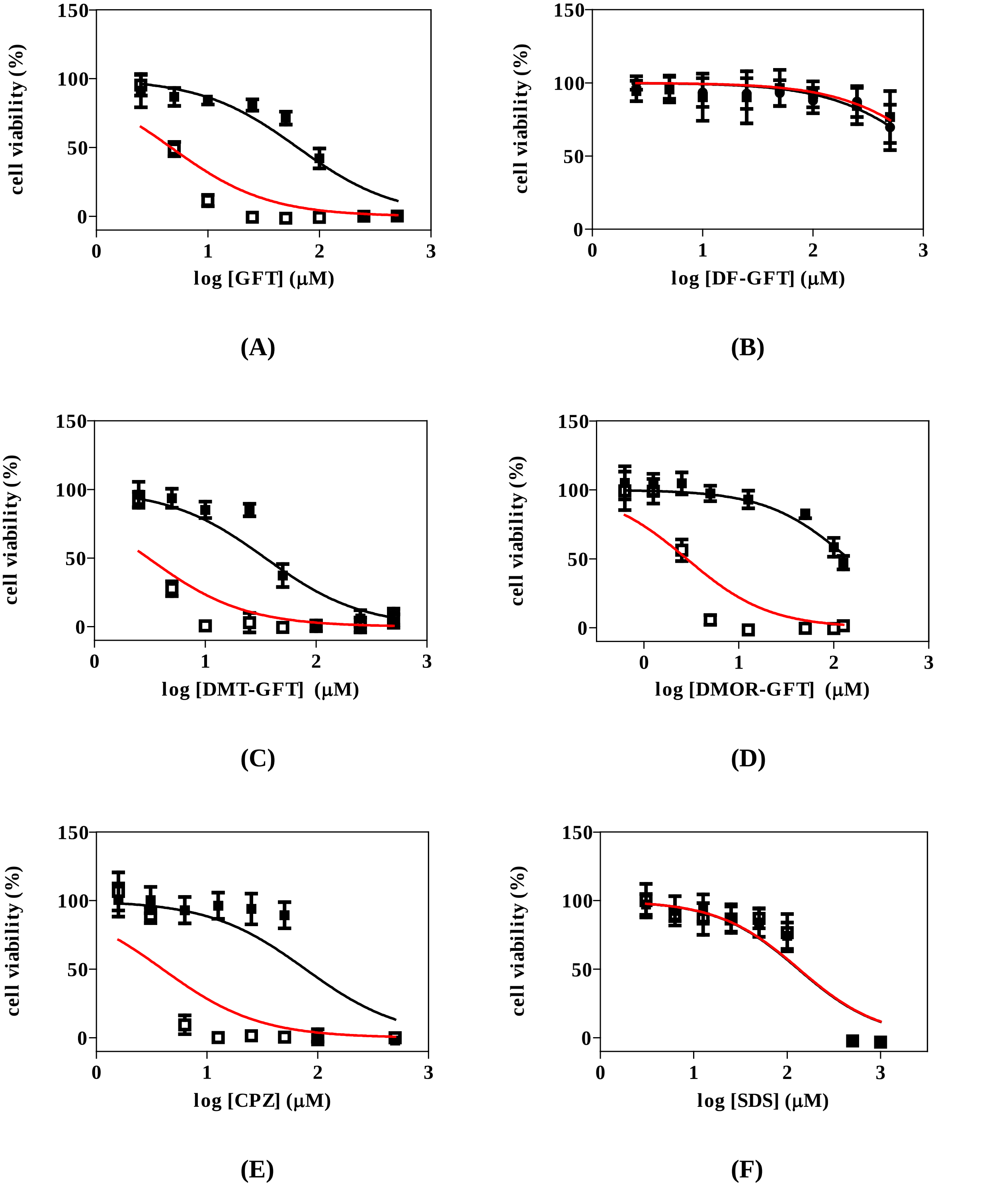

Supplementary Figure 1. Dose-response curves for cell viability of HaCaT cells treated with GFT **(A)**, GFT metabolites **(B-D)**, CPZ **(E)** and SDS **(F)** in the presence (□) or absence (■) of UVA Light (5 J/cm^2). Both CPZ and SDS were used as positive and negative phototoxicity controls, respectively. Data represent the mean ± SD from four independent experiments performed in triplicate.

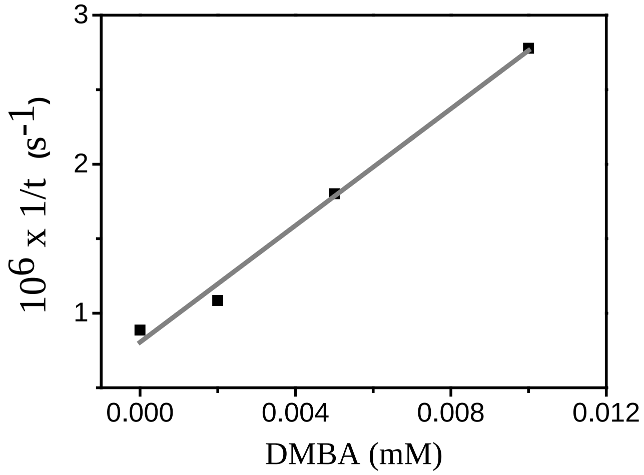

Supplementary Figure 2. Stern-Volmer plot obtained from triplet excited state lifetime (τ_T) measurements for DMOR-GFT after the addition of increasing amounts of quencher (DMBA).

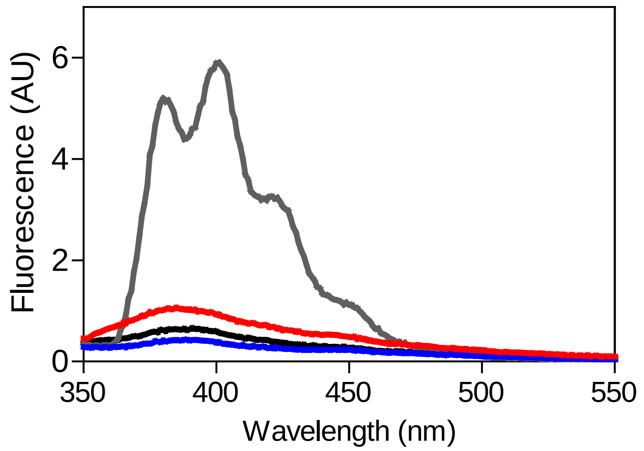

Supplementary Figure 3. Relative fluorescence emission of GFT (**black**), DMT-GFT (**blue**) and DMOR-GFT (**red**) in HaCaT cells after excitation at 320 nm. Anthracene (**gray**) was used as the standard for fluorescence quantum yield (Φ_F) measurements.

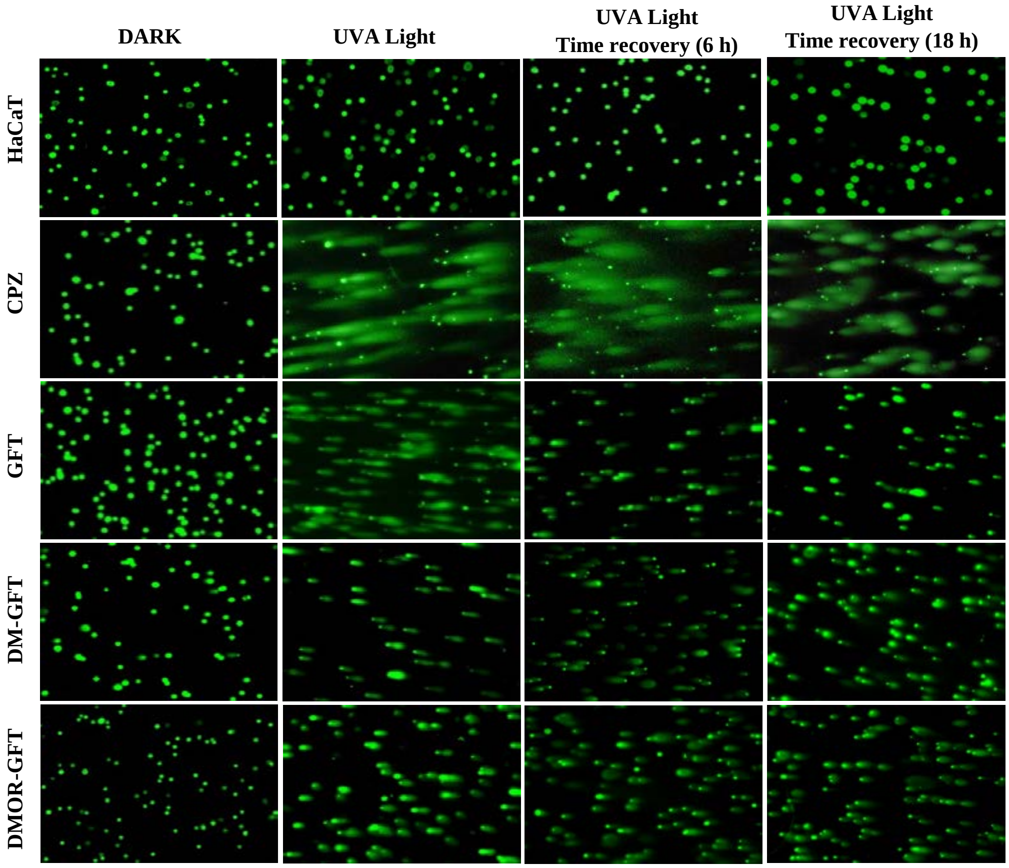

Supplementary Figure 4. Alkaline comet assay microscopy images of irradiated (UVA Light) or non-irradiated (Dark) cells non-treated or treated with GFT, DMT-GFT or DMOR-GFT and followed by 6 h or 18 h of cell recovery (UVA Light + Time recovery 6 h/18 h).
